# Regulation of P-glycoprotein by Bajijiasu *in vitro* and *in vivo* by activating the Nrf2-mediated signalling pathway

**DOI:** 10.1080/13880209.2019.1582679

**Published:** 2019-03-30

**Authors:** Xin Yang, Guoyan Hu, Lijuan Lv, Ting Liu, Longkai Qi, Guozhan Huang, Dongqing You, Jun Zhao

**Affiliations:** a The Fifth Affiliated Hospital of Guangzhou Medical University; The Fifth Clinical School of Guangzhou Medical University, Guangzhou, China;; b Department of Obstetrics and Gynecology, Guangdong Women and Children Hospital, Guangzhou, China;; c Guangdong Consun Pharmaceutical Group, Institute of Consun Co. for Chinese Medicine in Kidney Diseases, Guangzhou, China

**Keywords:** *Morinda officinalis* F.C. How, Keap1, transport protein, herb–drug interaction

## Abstract

**Context:** Bajijiasu (BJJS), a main bioactive compound from *Morinda officinalis* F.C. How. (Rubiaceae), is widely administered concomitantly with other drugs for treating male impotence, female infertility, fatigue, chronic rheumatism, depression, etc.

**Objective:** This study investigates the regulation of P-glycoprotein (P-gp) by BJJS *in vitro* and *in vivo*.

**Material and methods:** HepG2 cells were incubated with BJJS (10, 20 or 40 μM) for 48 h. C57 mice were orally treated with BJJS (25, 50 or 100 mg/kg) for 2 weeks. The protein and mRNA levels of P-gp were measured by using Western blot and real-time PCR, respectively. siNrf2 RNA was used to explore the mediation effects of Nrf2 on the P-gp expression. The efflux activity of P-gp was tested via a flow cytometry.

**Results:** Incubation of HepG2 cells with BJJS at 10, 20, and 40 μM up-regulated the P-gp protein expression by 12.3%, 82.9%, and 134.3%, respectively. Treatment of C57 mice with BJJS at 25, 50 and 100 mg/kg increased the P-gp protein expression by 49.3%, 75.8% and 106.0%, respectively. Incubation of the cells with BJJS at 10, 20 and 40 μM up-regulated the total Nrf2 protein levels by 34.3%, 93.1% and 118.6%, respectively, and also increased the nuclear Nrf2 protein levels by 14.8%, 44.4% and 59.25%, respectively. The total Nrf2 protein levels were increased by 46.3%, 66.5%, and 87.4%, respectively, in the mice exposed to BJJS at 25, 50, and 100 mg/kg. Inhibition of Nrf2 by siRNA diminished the P-gp induction by 25.0%, 33.4%, and 38.7%, respectively, in the cells. In addition, BJJS enhanced the efflux activity of P-gp by 9.6%, 37.1%, and 48.1%, respectively, in the cells.

**Conclusions:** BJJS activates Nrf2 to induce P-gp expression, and enhanced the efflux activity of P-gp. The possibility of potential herb–drug interactions when BJJS is co-administered with other P-gp substrate drugs should be carefully monitored.

## Introduction

**Figure 1.**
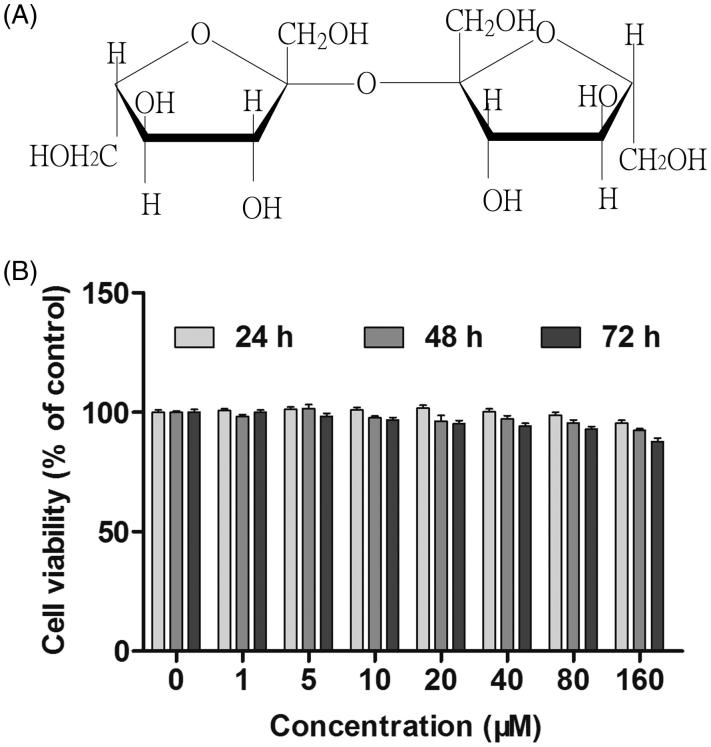
Chemical structure of BJJS (A) and effects of BJJS on viability of HepG2 cells (B). Cell viability was examined using the MTT assay. Data (% viability) are expressed as the mean ± SD (*n* = 6).

*Morinda officinalis* F.C. How. (Rubiaceae) (MO), also called ‘Bajitian’ in Chinese, is one of the best-known herbal drugs in China, Korea, and Japan. In traditional Chinese medicine (TCM), MO is frequently used to treat various diseases, including male impotence, female infertility, fatigue, chronic rheumatism, and depression (Song et al. 2015). Modern pharmacological experiments have indicated that MO possesses a wide range of pharmacological activities, including antioxidant, analgesic, anti-inflammatory, anti-osteoporosis, antidepressant, and pro-fertility activities (Li et al. 2009; Shi et al. 2015; Liang et al. 2017). MO has also been shown to contribute to improving memory and treating Alzheimer’s disease (AD) (Cai et al. 2017; Lee et al. 2017; Zhang et al. 2018). Bajijiasu (BJJS) (Figure 1(A)), which is one of the main bioactive compounds isolated from MO, possesses various pharmacological activities and therapeutic efficacies (Chen et al. 2014; Cai et al. 2017; Hong et al. 2017).

In Chinese clinical practice, multiherb therapy is one of the most important treatment methods to counteract toxicities or side effects and simultaneously enhance the therapeutic effects of the prescription herbs (Wu et al. 2014). MO is an herbal medicine commonly co-administered with other drugs for efficacy improvement. MO has been used as an essential ingredient in 103 Chinese traditional medicine preparations and 223 classic prescriptions for the treatment of chronic and complicated diseases (Zhang et al. 2018). More importantly, MO has been approved as a tonic or nutrient supplement that can be used in health food and functions to nourish the kidney, strengthen bones, and enhance immunity (Zhang et al. 2018). However, the potential herb–drug interactions (HDIs) have become a common clinical problem due to the increasing popularity of combination therapy between Chinese herbs and other drugs (Borrelli and Izzo 2009; Izzo and Ernst 2009). The high expression of P-glycoprotein (P-gp) induced by xenobiotic drugs during clinical treatment has been recognized by authorities as a key cause of HDIs (Wu et al. 2016). P-gp, encoded by MDR1, is an apical plasma member transport protein that is ubiquitously expressed in many vital organs and tissues in our body, such as the kidney, intestine, liver, lung, placenta, and blood–brain barrier cells (Giacomini et al. 2010). P-gp acts as an efflux pumps and functions to protect the human body against xenobiotics and cellular toxicants (Hu et al. 2014). Unfortunately, over-expression of P-gp can be easily induced after repeated exposure to numerous xenobiotic drugs, resulting in alterations in the toxicity and therapeutic response of the co-administered P-gp substrate drugs, thereby causing HDIs (Verschraagen et al. 1999; Zhou 2008). Currently, little is known about the impact of BJJS on P-gp. Whether potential HDIs may exist when BJJS is co-administered with P-gp substrate drugs have never been studied. Due to the wide co-administration of MO with other therapeutic drugs, some of which are also P-gp substrates, the study of P-gp regulation to assess potential HDIs resulting from the co-administration of BJJS and P-gp substrate drugs is essential.

Nuclear factor (erythroid-derived 2)-like 2 (Nrf2) signalling has been widely shown to be a key transcriptional pathway that regulates the expression of a number of cytoprotective genes (Shen and Kong 2009; He et al. 2014). The antioxidant response mediated by the Nrf2 pathway is the key cellular defence mechanism against the oxidative stress caused by xenobiotic exposure (Ma 2013). Under physiological conditions, Nrf2 is bound to its repressor Kelch-like ECH associating protein 1 (Keap1) in the cytosol. Upon exposure to xenobiotics, Nrf2 releases from Keap1, and translocates into the nucleus, where it binds to antioxidant response elements (AREs) in the promoter of numerous genes and promotes transcription of target genes (Ma 2013; He et al. 2014). Many studies have reported that Nrf2 plays a key role in the modulation of many drug transporters, including P-gp (Shen and Kong 2009; He et al. 2014). However, whether BJJS can regulate P-gp expression via activation of the Nrf2-mediated signalling pathway has not been explored as of yet. The change in P-gp expression may cause considerable changes in the pharmacokinetic profiles of substrate drugs (Wu et al. 2016). Therefore, it is important to identify the modulatory factors and signalling pathways involved in the regulation of P-gp by BJJS.

This study investigates the regulation of P-gp by BJJS *in vitro* and *in vivo*, and the regulatory mechanism involved. For this purpose, we first evaluated the effects of BJJS on the expression of P-gp in HepG2 cells and C57 mice. HepG2 cell line, which is established from human hepatic carcinoma, is a suitable *in vitro* model and has been widely used to study the regulation of P-gp expression and function of xenobiotic drugs (Rigalli et al. 2011; He et al. 2014). Second, we investigated whether BJJS can regulate P-gp expression via activation of the Nrf2-mediated signalling pathway. Third, we explored the effects of BJJS on the efflux activity of P-gp. Finally, we evaluated whether variation in the expression and activity of P-gp induced by BJJS can protect cells from the cytotoxicity produced by doxorubicin, a drug used in cancer chemotherapy and used here as a model P-gp substrate (Rigalli et al. 2011; Wu et al. 2016) The combined results of this study will help to predict the possibility of potential HDIs when BJJS is co-administered with other herbal medicines or chemical drugs that are P-gp substrates, thereby providing beneficial guidance for the correct application of MO in clinical practice.

## Materials and methods

### Chemicals and reagents

Rhodamine 123, doxorubicin, verapamil, and *tert*-butylhydroquinone (tBHQ) were purchased from Sigma-Aldrich (St. Louis, MO). Primary antibodies against human P-gp, Keap1 and Nrf2 were purchased from Abcam, Inc. (Cambridge, MA). Primary antibodies against human β-actin and histone H3, siNrf2, and siControl were obtained from Santa Cruz Biotechnologies Inc. (Santa Cruz, CA). Lipofectamine 2000 and TRIZOL were obtained from Invitrogen (Carlsbad, CA). All other chemicals and solvents used were of analytical reagent grade or better.

### Preparation of BJJS

BJJS (purity >98%) was provided by the School of Chinese Materia Medica of the Guangzhou University of Chinese medicine. BJJS was extracted from *Morinda officinalis* root according to a previous reference, and the purity of BJJS was analyzed using a high-performance liquid chromatography (HPLC) system (Chen et al. 2014). A voucher specimen (voucher no. 20161001) has been deposited in Department of Pharmacy, The Fifth Affiliated Hospital of Guangzhou Medical University (Guangzhou, China).

### Cell culture

HepG2 cells were obtained from the Shanghai Institute of Cell Biology, Chinese Academy of Sciences (Shanghai, China). The cells were maintained in DMEM supplemented with 10% (v/v) foetal bovine serum, 100 U/mL of penicillin, and 100 μg/mL of streptomycin in a humidified atmosphere of 5% CO_2_ and 95% air at 37 °C.

### Animals and treatments

Male C57 mice (20–22 g) were obtained from the Laboratory Animal Center of Guangdong Province (Guangzhou, China, SCXK, 2008-0020). Mice were reared at standard conditions within a free access to food and water. The mice were randomly divided into four groups (*n* = 8). The BJJS group orally received BJJS at 25, 50, or 100 mg/kg once per day over 2 weeks, respectively. The control group received only the same volume of the vehicle (water) for the same time period. Our research was approved by the Ethical Committee of Guangzhou University of Chinese Medicine (Guangzhou, China).

### MTT assay

The MTT assay was selected to evaluate the cytotoxicity of BJJS on HepG2 cells. Cells were cultured in 96-well plates for 24 h. After incubation with BJJS (0–160 μM) for 24, 48, or 72 h, the medium was removed, and MTT solution (100 μL, 0.5 mg/mL) was added to each well for another 4 h of incubation at 37 °C. At the end of the incubation, the medium was removed, and the purple formazan crystals were dissolved in 150 μL DMSO per well. The plates were read on a microplate reader at 570 nm. Cell viability ratio (%) = OD treated/OD control × 100%.

### Western blot analysis

HepG2 cells were seeded in six-well plates and exposed to BJJS (10, 20, and 40 μM) or the DMSO vehicle control for 48 h. As a positive control, HepG2 cells were incubated with tBHQ (10 μM, 48 h) for P-gp induction. For the isolation of total protein fractions, the medium was removed, and cells were washed twice with ice-cold PBS and lysed using RIPA lysis buffer supplemented with phenylmethylsulphonyl fluoride as a protease inhibitor. Isolation of nuclear fractions was performed as previously described (Seo et al. 2010). In the animal trial, the total proteins were extracted from the fresh livers of the sacrificed mice, and they were analyzed by Western blotting according to the published literature (He et al. 2014).

### Real-time PCR analysis

After the same treatment described above, the total RNA of HepG2 cells and mouse livers were extracted and reverse-transcribed to cDNA by using the TRIzol extraction method and a reverse transcription kit, respectively, according to the manufacturer’s instructions. Real-time PCR analysis of P-gp was performed according to the published literature (He et al. 2014). The sequences of the primers are listed in Supplementary Table 1.

### Transfection of siRNAs

For silencing experiments, HepG2 cells in the exponential growth phase were seeded in six-well plates and transfected with Nrf2-specific siRNA (siNrf2) or control siRNA (siCon) using Lipofectamine 2000 (Invitrogen, Carlsbad, CA) with optimized concentrations according to the manufacturer’s protocols. After 24 h, the efficiency of siRNA knockdown was assayed by real-time PCR analysis, as described above. Nrf2 primers were as follows: forward primer 5′-GGCATCACCAGAACACTCAG-3′ and reverse primer 5′-TGACCAGGACTTACAGGCAAT-3′. At the end of siRNA transfection, the cells were exposed to BJJS (10, 20, and 40 μM) for another 48 h. Cell lysates were collected by using RIPA lysis buffer supplemented with phenylmethylsulphonyl fluoride as a protease inhibitor, and were analyzed for the protein expression of P-gp by Western blot analysis as described above.

### Transport activity of P-gp

HepG2 cells were seeded in six-well plates and exposed to BJJS (10, 20, and 40 μM) or the DMSO vehicle control for 48 h. After incubations, the medium from the plates was removed, and fresh medium containing rhodamine 123 (2 μM), a typical P-gp substrate (Rigalli et al. 2011; Wu et al. 2016), was added. The plates were incubated for another 60 min at 37 °C. As positive control, HepG2 cells were incubated with verapamil (50 μM, 60 min) for inhibition of P-gp transport activity (Rigalli et al. 2011; Wu et al. 2016). At the end of rhodamine 123 incubation, the cells were washed twice with ice-cold PBS and resuspended in PBS. The fluorescence intensity of intracellular rhodamine 123 was detected by using a flow cytometer (excitation wavelength = 488 nm; emission wavelength = 535 nm). The mean fluorescence value was converted to a percentage of the control.

### Effects of BJJS against doxorubicin cytotoxicity

The antitumor drug doxorubicin, a P-gp substrate (Rigalli et al. 2011; Wu et al. 2016), was used to evaluate the effects of BJJS against doxorubicin cytotoxicity. After the same treatment with BJJS, the medium from the plates was removed, and fresh medium containing the antitumor drug doxorubicin (5 μM) was added. The plates were incubated for another 60 min at 37 °C. At the end of doxorubicin incubation, the cells were washed twice with ice-cold PBS and resuspended in PBS. The fluorescence intensity of intracellular doxorubicin was detected by using a flow cytometer (excitation wavelength = 488 nm; emission wavelength = 610 nm). The mean fluorescence value was converted to a percentage of the control.

The MTT assay was also used to test the potential role of BJJS against the cytotoxicity produced by doxorubicin. HepG2 cells were seeded in 96-well plates and pretreated with BJJS (10, 20, and 40 μM) or the DMSO vehicle control for 48 h. After the treatment, the medium was replaced with fresh medium containing different concentrations of doxorubicin (0–6.4 μg/mL) for an additional 48 h incubation. Cell viability was evaluated using the MTT assay. Cytotoxicity was expressed as the concentration of doxorubicin inhibiting cell growth by 50% (IC_50_).

### Data analysis

All results were expressed as the mean values ± standard deviation (SD) from three independent experiments. The significance of the difference was calculated by using Student’s *t*-test or one-way ANOVA by SPSS 19.0 software (SPSS, Chicago, IL). Values of *p* < 0.05 were considered significant.

## Results

### Effects of BJJS on HepG2 cell viability

The cell viability was examined using the MTT assay. Figure 1(B) shows the cytotoxicity of BJJS on HepG2 cells. No significant change in cell viability was observed when cells were treated with BJJS at 0–160 µM for 24–72 h. According to these results, the concentrations of 10, 20, and 40 μM and a 48 h treatment period were selected to perform subsequent experiments. At these treatment conditions, the cell viability was over 85%.

### Regulation of P-gp protein and mRNA levels by BJJS in HepG2 cells

**Figure 2.**
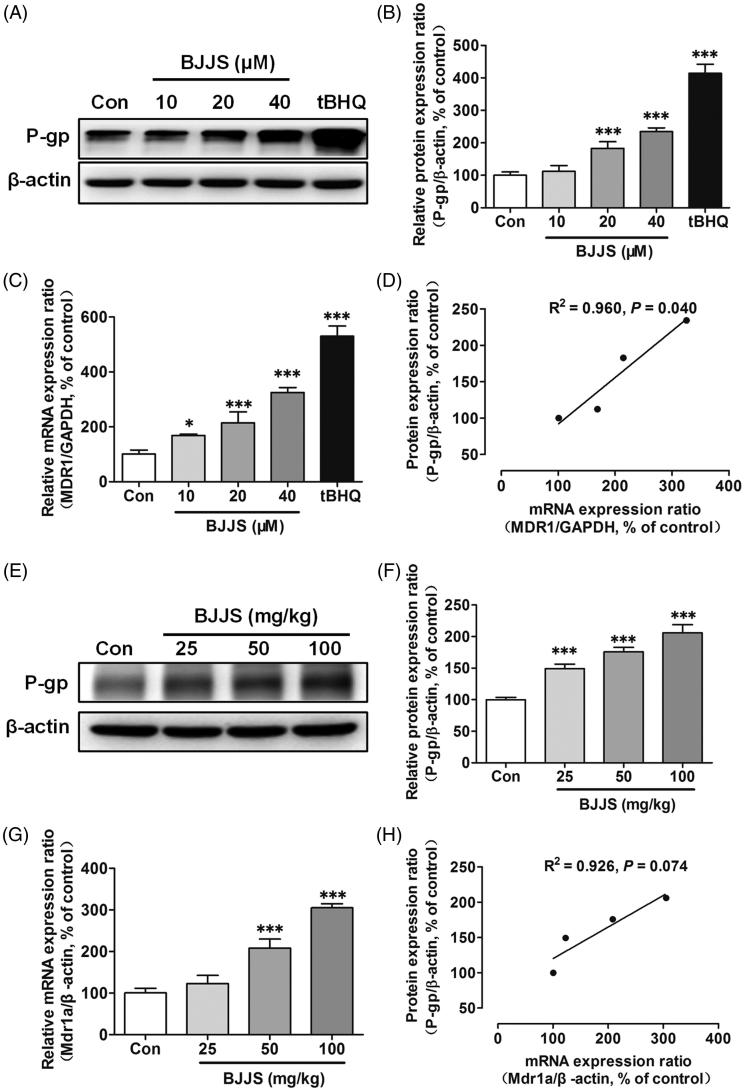
Effects of BJJS on protein and mRNA levels of P-gp in HepG2 cells (A, B, and C) and C57 mice (E, F, and G). Protein and mRNA expression levels were measured using Western blot and real-time PCR analysis, respectively. The correlation between P-gp mRNA levels and protein levels in HepG2 cells (D) and C57 mice (H) was analyzed using Person analysis, respectively. Densitometry results were related to β-actin and are presented as a percentage of control. GAPDH was used as a housekeeping gene for cells. Data are represented as the mean ± SD (*n* = 3). *p < 0.05 and ***p < 0.001 compared with the control group by using one-way ANOVA analysis.

The protein and mRNA expression levels of P-gp were evaluated by Western blot and real-time PCR analysis, respectively. Figure 2(A,B) shows the regulation of the protein levels of P-gp by BJJS. Compared to the levels in the control group, the protein levels of P-gp were significantly (*p* < 0.001) higher in the treatment groups given 20 and 40 μM of BJJS for 48 h. A marked increase in P-gp levels (*p* < 0.001) was also observed when tBHQ (10 μM), a positive control for P-gp induction, was used to treat the HepG2 cells. Figure 2(C) shows the regulation of the P-gp mRNA levels by BJJS. The P-gp mRNA levels in the cells exposed to the same treatment were also significantly (*p* < 0.05 or *p* < 0.001) higher than in the control cells. The P-gp mRNA levels were markedly elevated in the cells exposed to tBHQ (10 μM). The correlation between P-gp mRNA levels and protein levels was analyzed by using Person analysis. The result showed that the increase in P-gp mRNA expression was closely (*r*^2^ = 0.960. *p* < 0.05) related to that in P-gp protein levels induced by BJJS (Figure 2(D)).

### Regulation of P-gp protein and mRNA levels by BJJS in C57 mice

Figure 2(E,F) shows the regulation of the P-gp protein levels by BJJS in C57 mice. Compared to the levels in the control group, the protein levels of P-gp were markedly (*p* < 0.001) higher in the treatment groups given 25, 50, and 100 mg/kg of BJJS for 2 weeks. Figure 2(G) shows the regulation of the mRNA levels of Mdr1a by BJJS. The Mdr1a mRNA levels in the mice exposed to the same treatment were also strikingly higher than in the control mice. Person analysis showed that the increase in Mdr1a mRNA expression was also closely (*r*^2^ = 0.926) related to that in P-gp protein levels induced by BJJS (Figure 2(H)).

### Nuclear translocation of Nrf2 by BJJS in HepG2 cells

**Figure 3.**
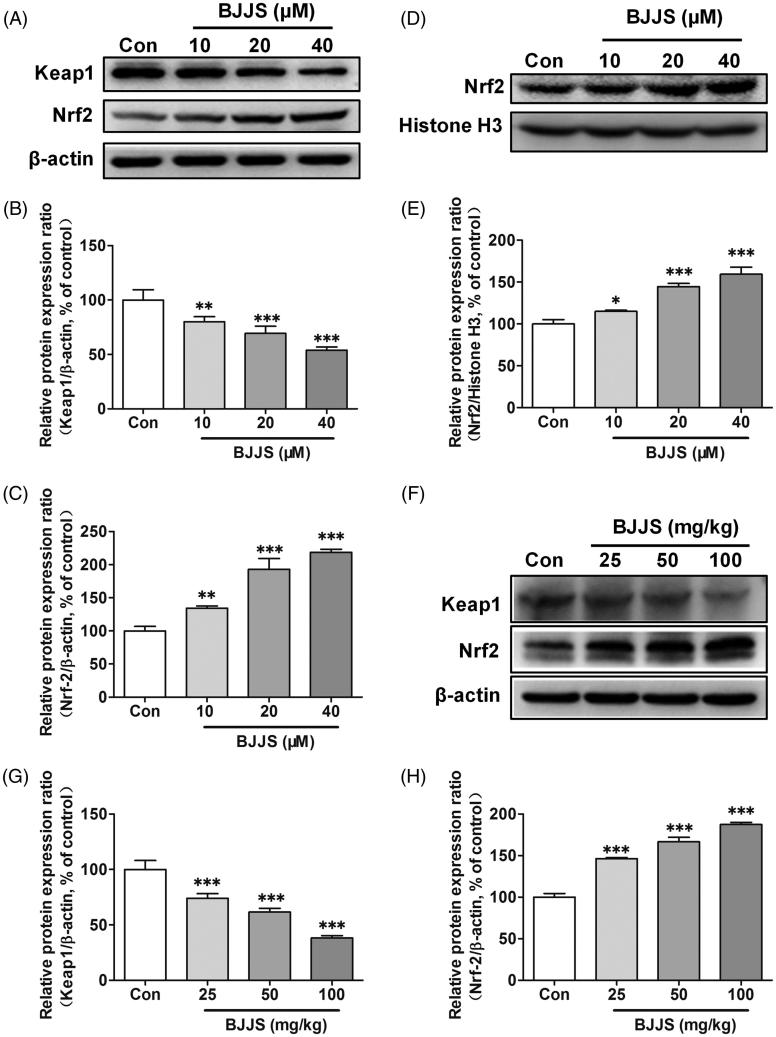
Effects of BJJS on the protein levels of Keap1 (A and B), total Nrf2 (A and C), and nuclear Nrf2 (D and E) in HepG2 cells. Effects of BJJS on the protein levels of Keap1 (F and G), and Nrf2 (F and H) in C57 mice. Protein expression levels were measured by using Western blot analysis. Densitometry results were related to β-actin and presented as the percentage of control. Data are represented as the mean ± SD (*n* = 3). **p < 0.01 and ***p < 0.001 compared with the control group by using one-way ANOVA analysis.

The protein expression levels of Nrf2 and Keap1 were evaluated by Western blot analysis. Compared to the levels in the control cells, the Keap1 protein levels in the HepG2 cells exposed to 10, 20, and 40 μM of BJJS were markedly (*p* < 0.01 or *p* < 0.001) decreased (Figure 3(A,B)). The total Nrf2 protein levels in the cells with the same treatment were significantly (*p* < 0.01 or *p* < 0.001) increased (Figure 3(A,C)). A strong negative correlation (*r*^2^ = −0.979, *p* < 0.05) was observed between MDR1 mRNA levels and Keap1 protein levels (Supplementary Figure 1(A)). A strong positive correlation (*r*^2^ = 0.951, *p* < 0.05) was also observed between MDR1 mRNA levels and Nrf2 protein levels (Supplementary Figure 1(B)). The nuclear Nrf2 protein levels were also evaluated by Western blot analysis. As shown in Figure 3(D,E), exposure to the same concentrations of BJJS resulted in markedly (*p* < 0.05 or *p* < 0.001) increases in nuclear Nrf2 protein levels compared with control treatment. Person analysis showed that P-gp mRNA expression was also closely (*r*^2^ = 0.957, *p* < 0.05) related to nuclear Nrf2 protein levels (Supplementary Figure 1(C)).

### Regulation of Nrf2 signalling pathway by BJJS in C57 mice

The keap1 and Nrf2 levels in the liver of mice exposed to BJJS were evaluated by Western blot analysis. Compared to the control mice, the treatment of BJJS (25, 50, and 100 mg/kg) for 2 weeks significantly (*p* < 0.001) decreased the keap1 protein levels in a dose-dependent manner (Figure 3(F,G)). The Nrf2 protein levels in the mice with the same treatment were significantly (*p* < 0.001) increased (Figure 3(F,H)). A closely negative correlation (*r*^2^ = −0.947) was observed between Mdr1a mRNA levels and Keap1 protein levels (Supplementary Figure 2(A)). A positive correlation (*r*^2^ = 0.895) was also observed between Mdr1a mRNA levels and Nrf2 protein levels (Supplementary Figure 2(B)).

### Nrf2-mediated P-gp induction by BJJS

**Figure 4.**
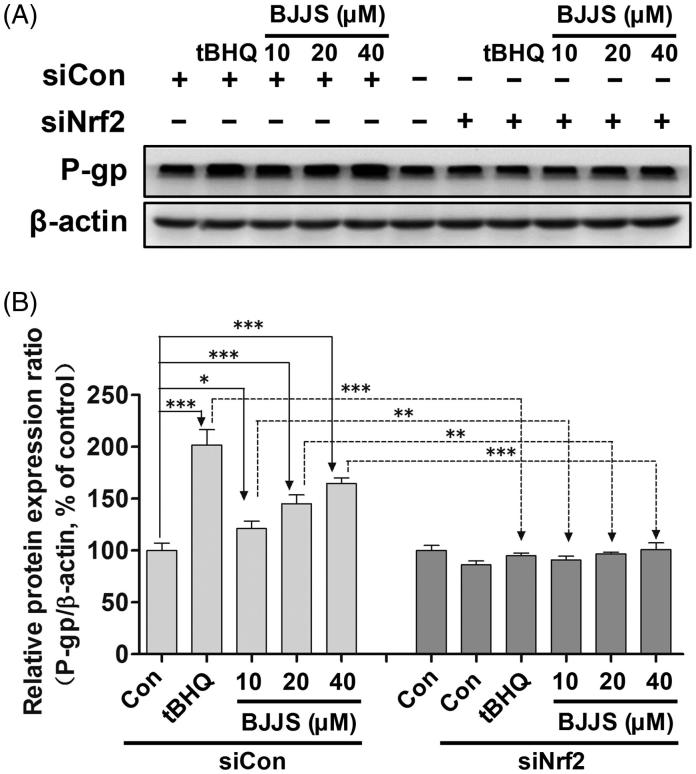
Mediation effects of Nrf2 on P-gp expression induced by BJJS. HepG2 cells were transfected with Nrf2-specific siRNA (siNrf2) or control siRNA (siCon). Protein levels of P-gp were measured using Western blot analysis. Densitometry results were related to β-actin and presented as percentage of control. Data are represented as the mean ± SD (*n* = 3). **p < 0.01 and ***p < 0.001 compared to the control group by using one-way ANOVA analysis.

To explore the mediation effects of Nrf2 on the P-gp expression induced by BJJS, Nrf2 expression was silenced in cells. Cells transfected with siNrf2 RNA showed a significant (*p* < 0.001) reduction in Nrf2 expression compared to cells transfected with the control siRNA (Supplementary Figure 3). As shown in Figure 4(A,B), siNrf2 RNA significantly suppressed the BJJS-enhanced P-gp protein levels, whereas the control siRNA did not suppress the expression levels of these proteins. In addition, siNrf2 RNA significantly (*p* < 0.001) prevented increases in P-gp protein levels induced by tBHQ.

### Regulation of the efflux activity of P-gp by BJJS

**Figure 5.**
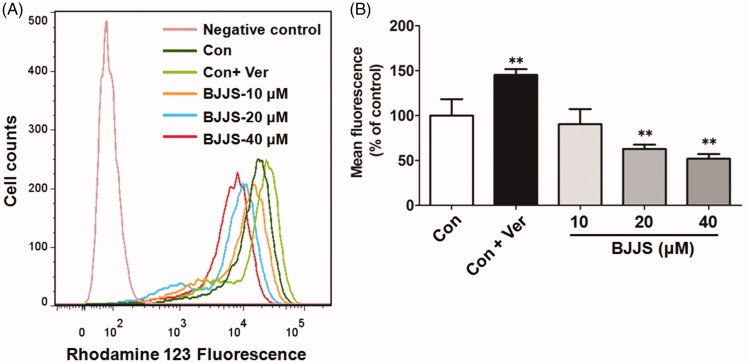
Effects of BJJS on transport activity of P-gp. Rhodamine 123, a typical P-gp substrate, was selected as the probe substrate to evaluate the efflux activity of P-gp. Rhodamine 123 accumulation in HepG2 cells was measured by flow cytometry. Data are expressed as percentage of control and represent the mean ± SD (*n* = 3). **p < 0.01 compared with the control group by using one-way ANOVA analysis.

Rhodamine 123, a typical P-gp substrate, was selected as a probe substrate to evaluate the efflux activity of P-gp. As shown in Figure 5, BJJS (10, 20, or 40 μM) treatment for 48 h in HepG2 cells dose-dependently decreased intracellular rhodamine 123 accumulation, compared to control treatment. Ver, a positive drug for inhibition of P-gp transport activity, significantly (*p* < 0.01) increased rhodamine 123 accumulation in cells.

### Effects of BJJS against doxorubicin cytotoxicity

**Figure 6.**
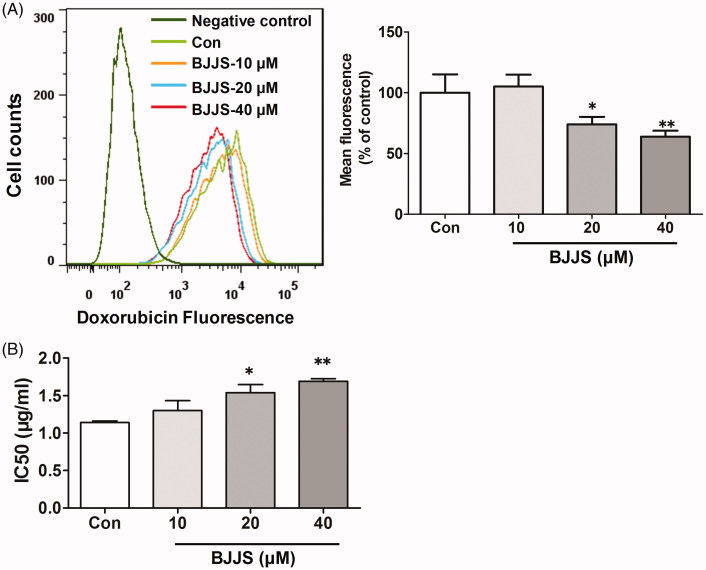
Effects of BJJS against doxorubicin cytotoxicity. The antitumor drug doxorubicin (a P-gp substrate) was used to evaluate the effects of BJJS against doxorubicin cytotoxicity. Doxorubicin accumulation in HepG2 cells was measured by flow cytometry (A). Cell viability was evaluated by the MTT assay. Cytotoxicity was expressed as the concentration of doxorubicin inhibiting cell growth by 50% (IC_50_) (B). Data shown represent the mean ± SD (*n* = 3). *p < 0.05 and **p < 0.01 compared with the control group by using one-way ANOVA analysis.

Figure 6 shows the protective effects of BJJS treatment (10, 20, or 40 μM, 48 h) against the cytoxicity of the antitumor drug doxorubicin (a substrate of P-gp) towards HepG2 cells. Intracellular doxorubicin accumulation in the BJJS groups was significantly lower (*p* < 0.05 or *p* < 0.05, respectively) than in the control group (Figure 6(A)). In addition, the same incubation of HepG2 cells significantly (*p* < 0.01 or *p* < 0.05, respectively) increased the IC_50_ value related to doxorubicin cytotoxicity (Figure 6(B)).

## Discussion

In Chinese clinical practice, MO is frequently used concomitantly with other herbal medicines or chemical drugs to treat complex diseases (Zhang et al. 2018). This herb is also popularly consumed as a food additive to nourish the kidney and reinforce bodily immunity (Zhang et al. 2018). Thus, the current study assessed the potential HDIs caused by co-administration of MO with other drugs.

BJJS is one of the main bioactive compounds from MO and possesses various pharmacological activities and therapeutic efficacies (Chen et al. 2014; Cai et al. 2017; Hong et al. 2017). The HepG2 cell line, which is established from human hepatic carcinoma, is a suitable *in vitro* model and has been widely used to study the regulation of P-gp expression and function by xenobiotic drugs (Rigalli et al. 2011; He et al. 2014). Using the MTT assay, we confirmed that the treatment of BJJS at 0–160 µM for 24–72 h did not elicit remarkable cytotoxicity toward HepG2 cells (Figure 1(B)). Accordingly, concentrations of 10, 20, and 40 μM and a 48 h treatment period were selected to perform subsequent experiments.

We observed that BJJS could significantly induce the protein expression of P-gp in a dose-dependent manner in HepG2 cells (Figure 2(A,B)). A marked increase in P-gp protein levels was also observed for the positive control tBHQ, a well-known potent Nrf2 activator (Seo et al. 2010; He et al. 2014). To determine whether the up-regulation of P-gp protein expression was due to increased mRNA expression, we measured the MDR1 mRNA levels and found that the same treatment of BJJS also produced a significant increase in MDR1 mRNA levels, consistent with the change in P-gp protein levels (Figure 2(C)). Person analysis showed that the increase in P-gp mRNA levels was closely related to that in P-gp protein levels, suggesting that BJJS regulated the transcription of P-gp (Figure 2(D)). C57 mice were further selected to confirm the induction of P-gp by BJJS *in vivo*. We observed that BJJS could also markedly induce the protein and mRNA levels of P-gp in a dose-dependent manner (Figure 2(E–G)). The close positive correlation between the P-gp protein and mRNA expression further implied that BJJS regulated the transcription of P-gp (Figure 2(H)). The high expression of P-gp induced by xenobiotic drugs during the clinical treatment has been recognized as one of the key causes of HDIs (Verschraagen et al. 1999; Zhou 2008; Klaassen and Aleksunes 2010; Wu et al. 2016). Therefore, we speculated that the potential HDIs likely occurred when BJJS was used concomitantly with other P-gp substrate drugs.

Nrf2 signalling is considered to be a key transcriptional pathway that regulates the expression of a number of transporters, including P-gp (Shen and Kong 2009; He et al. 2014). Upon exposure to xenobiotics, Nrf2 dissociates from Keap1, a Nrf2 repressor that binds Nrf2 in the cytoplasm, followed by nuclear translocation. In the nucleus, Nrf2 heterodimerizes with other partner proteins and binds to AREs in the promoter of the target genes (Shen and Kong 2009; Ma 2013). It is evident that many bioactive compounds from herbal drugs can activate the Nrf2 signalling pathway, leading to up-regulation of P-gp (He et al. 2014; Hou et al. 2018). Thus, we further examined the effect of BJJS on Keap1 expression and the nuclear translocation of Nrf2 to clarify the role of the Nrf2-mediated signalling pathway in the induced effects of BJJS on P-gp. We observed that incubation of HepG2 cells with BJJS dose-dependently decreased the cytosolic level of Keap1 (Figure 3(A,B)), and increased the total Nrf2 expression (Figure 3(A,C)). A strong negative correlation was observed between MDR1 mRNA induction and increased Keap1 protein levels (Supplementary Figure 1(A)). A strong positive correlation was also found between MDR1 mRNA induction and the increase in Nrf2 protein levels (Supplementary Figure 1(B)). We also examined the effect of BJJS on Nrf2 protein expression in the nucleus. The results showed that BJJS significantly up-regulated Nrf2 protein levels in the nucleus in a dose-dependent manner (Figure 3(D,E)). MDR1 mRNA induction was closely related to the nuclear Nrf2 protein levels (Supplementary Figure 1(C)). We also observed that BJJS dose-dependently decreased the Keap1 level (Figure 3(G,G)), and increased the Nrf2 levels in the liver of C57 mice (Figure 3(F,H)). A strong correlation between Mdr1a levels and Keap1 (Supplementary Figure 2(A)), or Nrf2 (Supplementary Figure 2(B)) protein levels was also found. These results suggested that BJJS could facilitate the translocation of Nrf2 from the cytoplasm to the nucleus in HepG2 cells, thereby activating the expression of P-gp. To further confirm the mediation effects of Nrf2 on P-gp expression induction by BJJS, Nrf2 was silenced in cells (Supplementary Figure 3). We observed that compared with the control siRNA, siNrf2 RNA significantly suppressed BJJS-enhanced P-gp protein expression (Figure 4). Taken together, these results suggested that BJJS induced P-gp expression via the activation of the Nrf2 signalling pathway.

P-gp acts as a key efflux pump to protect the human body by pumping external chemicals out of cells (Giacomini et al. 2010; Hu et al. 2014). Thus, P-gp efflux activity was further evaluated by the ability of the HepG2 cells to export rhodamine 123, a typical P-gp substrate (Rigalli et al. 2011; Wu et al. 2016). As shown in Figure 5, the exposure of the cells to BJJS significantly decreased the intracellular content of rhodamine 123 in a dose-dependent manner, suggesting that the same BJJS treatment resulted in a significant increase in P-gp efflux activity. Obviously, the increase in P-gp efflux activity corresponded to the induction in P-gp expression by BJJS. These combined data indicate that clinical co-application of BJJS with other P-gp substrate drugs likely caused potential P-gp-mediated HDIs.

Widespread expression of P-gp in prominent bodily organs not only has protective function against some external chemicals but also affects the pharmacokinetics of many substrate chemotherapeutic drugs (Allen et al. 2002). Extensive studies and reviews have reported that high expression of P-gp can mediate the efflux of cytotoxic drugs out of cancer cells, leading to multidrug resistance and chemotherapy failure (Eckford and Sharom 2009; Wu et al. 2016). Thus, we postulated that BJJS could decrease the toxicity produced by chemotherapeutic agents towards HepG2 cells through induction of P-gp. We observed that the intracellular accumulation of doxorubicin, a P-gp substrate anticancer drug (Rigalli et al. 2011; Wu et al. 2016), in the cells exposed to BJJS was significantly lower than in the control cells (Figure 6(A)). In addition, the same incubation significantly increased the IC_50_ values related to doxorubicin cytotoxicity relative to control cells (Figure 6(B)). Taken together, these results indicate that BJJS could decrease the cytotoxicity of doxorubicin towards the cells.

In summary, the current studies demonstrated that BJJS, a bioactive compound from MO, up-regulated the expression of P-gp via activation of the Nrf2-mediated signalling pathway in HepG2 cells. BJJS also enhanced the efflux activity of P-gp, concomitantly decreasing the toxicity of doxorubicin towards cells. Due to the wide co-use of MO with other therapeutic drugs and because some of these drugs are also P-gp substrates, the possibility of potential HDIs when BJJS is co-administered with other P-gp substrate drugs should be carefully monitored in clinics.

## Supplementary Material

Supplemental Material
