# Supplementary material for: Regulation of P-glycoprotein by Bajijiasu in vitro and in vivo by activating the Nrf2-mediated signalling pathway
Source: Pharm Biol. 2019 Mar 30;57(1):184–92. doi: 10.1080/13880209.2019.1582679 (PMC6450468; doi:10.1080/13880209.2019.1582679)
Supplement: Supplemental Material [file IPHB_A_1582679_SM8059.docx]

**Supplementary Legends**

**Supplementary Figure 1.** Pairwise correlation between MDR1 mRNA levels and Keap1 (A), total Nrf2 (B) or nuclear Nrf2 (C) protein levels in HepG2 cells. The correlations were analyzed by using Person analysis.

**Supplementary Figure 2.** Pairwise correlation between Mdr1a mRNA levels and Keap1 (A), or Nrf2 (B) protein levels in C57 mice. The correlations were analyzed by using Person analysis.

**Supplementary Figure 3.** The efficiency of Nrf2 silencing. The mRNA expression levels of Nrf2 were evaluated by using real-time PCR analysis. Data shown represent the mean ± SD (*n* = 3). ^***^*p* < 0.001 compared with the siCon group by using Student’s *t*-test analysis.

**Supplementary Figure 1**

**
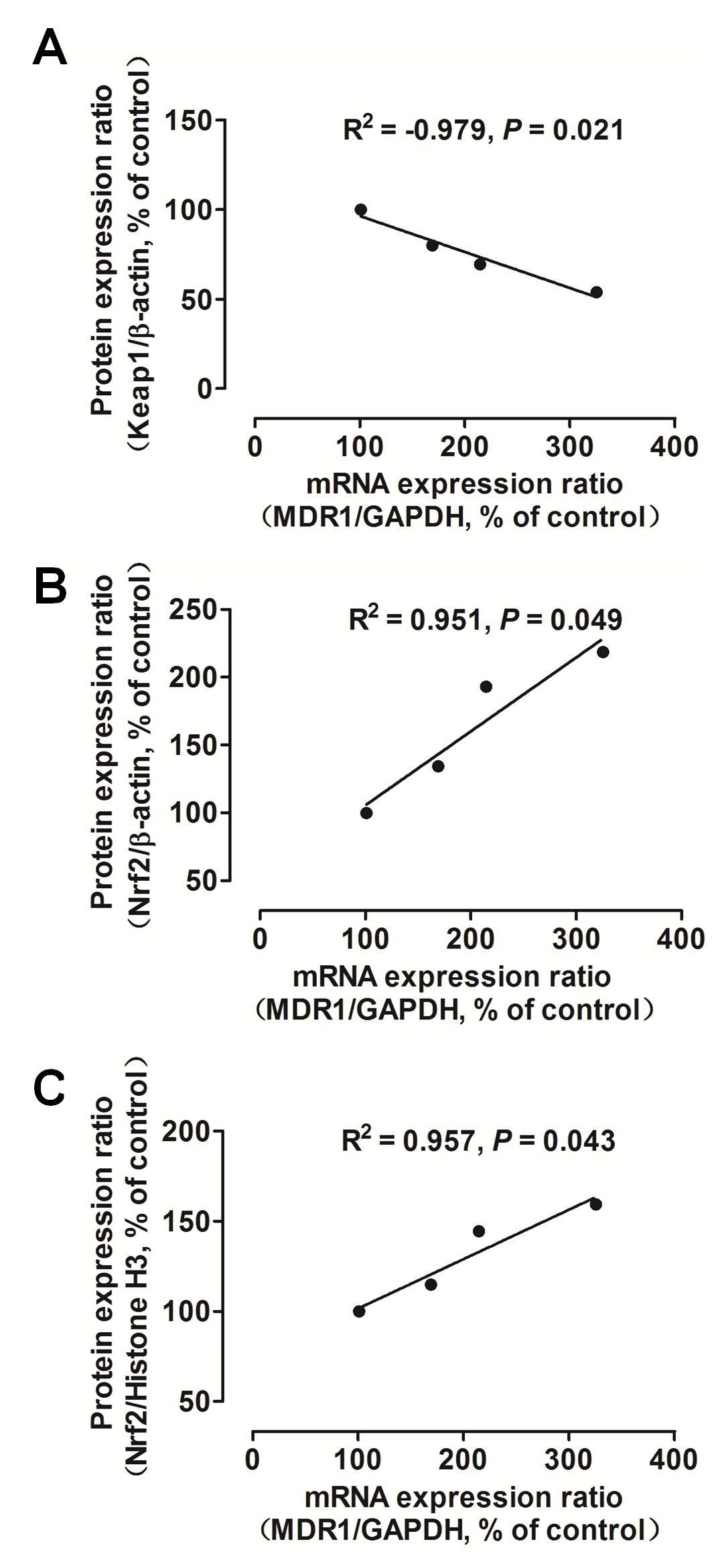
**

**Supplementary Figure 2**

**
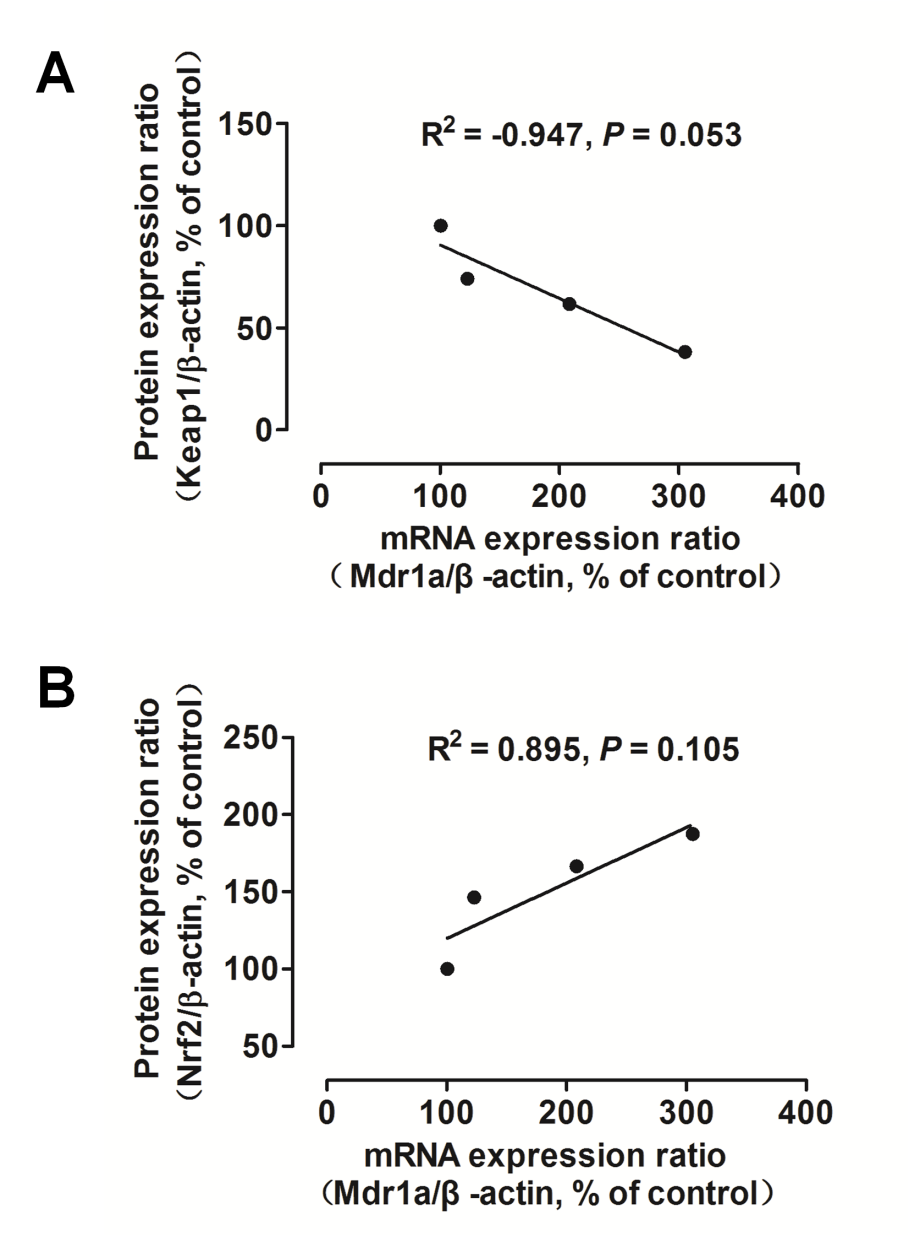
**

**Supplementary Figure 3**

**
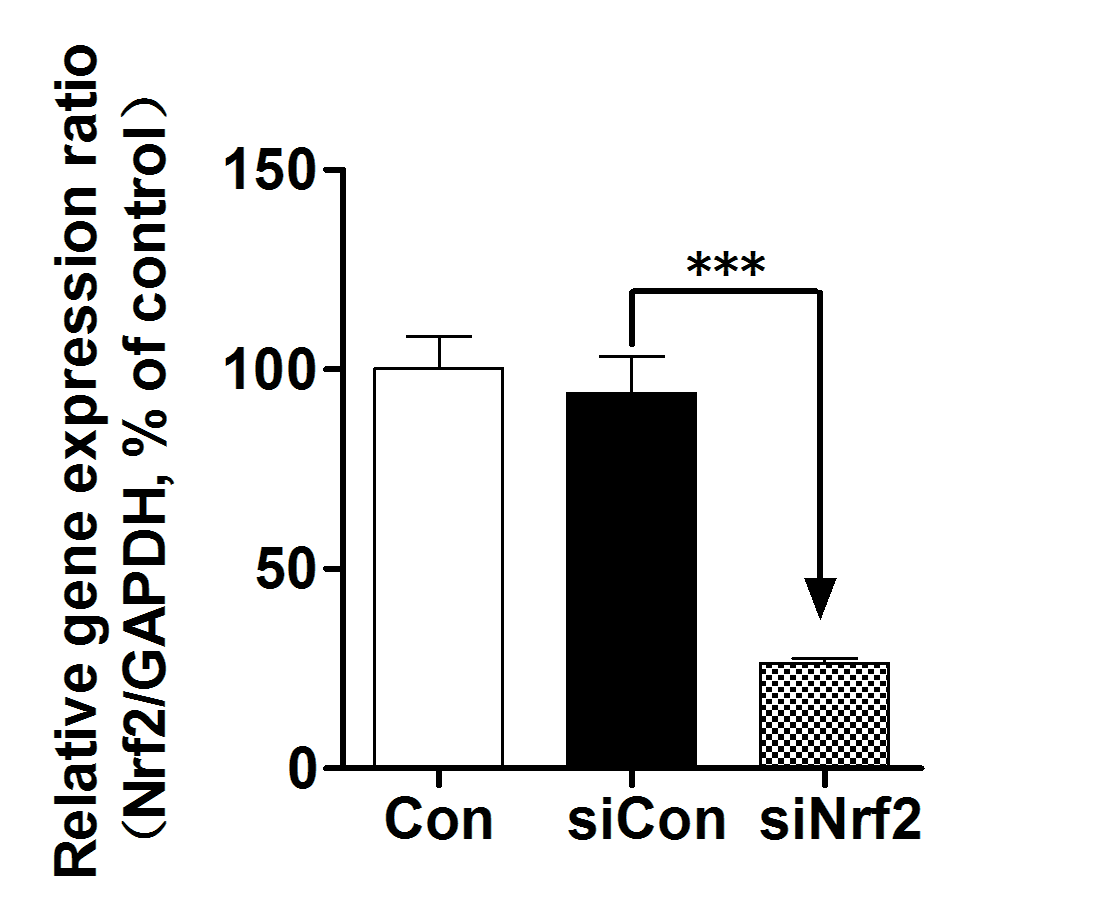
**

**Supplementary Table 1**. Gene-specific polymerase chain reaction primers.

| Gene | Forward (5′-3′) | Reverse (5′-3′) | Length (bp) |
| --- | --- | --- | --- |
| Mdr1a (mouse, NM_011076) | CATGACAGATAGCTTTGCAAGTGTAG | GGCAAACATGGCTCTTTTATCG | 85 |
| β-actin (mouse, NM_007393) | GGCTGTATTCCCCTCCATCG | CCAGTTGGTAACAATGCCATGT | 154 |
| MDR1 (human, NM_000927) | TGCTCAGACAGGATGTGAGTTG | AATTACAGCAAGCCTGGAACC | 122 |
| GAPDH (human, NM_001256799) | GGCCTCCAAGGAGTAAGACC | AGGGGAGATTCAGTGTGGTG | 122 |
